# Patient-Related Risk Factors for Chemotherapy-Induced Nausea and Vomiting: A Systematic Review

**DOI:** 10.3389/fphar.2020.00329

**Published:** 2020-04-01

**Authors:** Abu Saleh Mohammad Mosa, A. Mosharraf Hossain, Beau James Lavoie, Illhoi Yoo

**Affiliations:** ^1^Department of Health Management and Informatics, School of Medicine, University of Missouri, Columbia, MO, United States; ^2^Informatics Institute, University of Missouri, Columbia, MO, United States; ^3^Institute for Clinical and Translational Science, School of Medicine, University of Missouri, Columbia, MO, United States; ^4^Division of Hematology and Medical Oncology, School of Medicine, University of Missouri, Columbia, MO, United States

**Keywords:** cancer, Chemotherapy-Induced Nausea and Vomiting (CINV), patient-related risk factors, systematic review, emetogenicity

## Abstract

**Background:**

Studies have reported that patient-related factors significantly impact the risk of Chemotherapy-Induced Nausea and Vomiting (CINV). The objective of this study was to analyze those risk factors of CINV through a systematic literature review.

**Methods:**

We searched MEDLINE to identify articles that addressed patient-related risk factors of CINV through clinical studies.

**Results:**

A total of 49 articles were selected for this study. A total of 28 patient-related risk-factors that significantly impact the risk of CINV were documented. Three factors are demographically related, 17 factors are intrinsic in nature and innate to patient's physiology or influenced by physiology, and eight factors are extrinsic in nature. At least five studies identified seven risk factors with notable summary odds ratio: history of nausea/vomiting (odds ratio: 3.13, 95% CI 2.40–4.07, p < 0.05), female sex (odds ratio: 2.79, 95% CI 2.26–3.44, p < 0.05), expectancy of CINV (odds ratio: 2.61, 95%CI 1.69–4.02, p < 0.05), younger age (odds ratio: 2.59, 95% CI 2.18–3.07, p < 0.05), anxiety (odds ratio: 2.57, 95% CI 1.94–3.40, p < 0.05), history of morning sickness (odds ratio: 1.97, 95% CI 1.46–2.65, p < 0.05), and low alcohol intake (odds ratio: 1.94, 95% CI 1.68–2.24, p < 0.05).

**Conclusions:**

Oncologists can use these factors prior to the initiation of a chemotherapy regimen to identify patients at risk for CINV, in order to focus on more comprehensive antiemetic treatment options for those high-risk patients. This may enable better outcomes and avoid complications.

## Introduction

Chemotherapy is a core component of nearly every cancer treatment plan. Hawkins and Grunberg (2009) reported that as many as one million Americans receive chemotherapy each year. Chemotherapy causes many side-effects. Chemotherapy-Induced Nausea and Vomiting (CINV) is one of the most unpleasant and feared side effects of chemotherapy (Sun et al., 2005). There are several antiemetic guidelines for managing CINV. The guideline-recommended standard antiemetic prophylaxis for CINV considers only the emetogenicity of the chemotherapeutic agents. The emetogenicity of chemotherapy is divided into four emetic risk categories: (1) minimal, (2) low, (3) moderate, and (4) high. These four categories are divided based on the percentages of patients who suffer from CINV without antiemetics: (1) minimal emetogenetic chemotherapies have less than 10% risks, (2) low emetogenetic chemotherapies (LEC) have 10% to 30% risks, (3) moderate emetogenetic chemotherapies (MEC) have 30% to 90% risks, and (4) high emetogenetic chemotherapies (HEC) have more than 90% risks.

Several patient-related factors affect the risk of CINV. However, none of the guidelines considers those factors except the NCCN guidelines suggesting that regimens be chosen based on the drug with highest emetic risk as well as patient-specific risk factors (NCCN Clinical Practice Guidelines in Oncology: Antiemesis, Version 2.2015, 2015). As seen in the emetic risk categories, not all patients have the similar emetic risk of CINV. Despite improvements in CINV management, as many as two-thirds of patients still experience some degree of CINV. Several recent studies reported various percentages of CINV occurrence with use of antiemetics: from 28% at best to 62% at worst [28% (Jones et al., 2011), 38%–52% (Glaus et al., 2004), 56.1% (Molassiotis et al., 2008), 61.2% (Haiderali et al., 2011), and 62% (Cohen et al., 2007)]. As a result, physicians use their personal experiences with the treatment of CINV, which leads to inconsistent management of CINV.

There is a lack of comprehensive review of the literature and meta-analysis for summarizing the patient-related risk factors of CINV. Warr (Warr, 2014) summarized seven patient-related risk factors that had been found in at least two clinical trials of substantial size. The author noted that “the number of patient characteristics found in at least univariate analysis to influence the chance of emesis is sufficiently large that it is not possible to list all of them.” Most of the CINV-related review articles focused on the review of guidelines and review of CINV prophylaxis (Natale, 2015; Adel, 2017; Jordan et al., 2017).

The objective of this study was to identify patient-related factors that significantly impact the risk of CINV by conducting a systematic review of the literature. The reporting of this systematic review follows the “Preferred Reporting Items for Systematic Reviews and Meta-Analyses (PRISMA)” guideline (Moher et al., 2009).

## Methods

### Data Sources

In June 2019, we used PubMed to search the MEDLINE database for eligible articles. The search terms are related to the following terms: antiemetics, CINV and risk factors. We used a comprehensive search strategy to ensure retrieval of all relevant documents in MEDLINE. **Table 1** presents the entire search strategy.

**Table 1:** PubMed Search Strategy.

**Search Topic**	**Search Strategy**
**Antiemetics**	1. “antiemetics”[All Fields] 2. “antiemetics”[MeSH Terms] 3. “antiemetics”[Pharmacological Action] 4. OR (1,2,3)
**CINV**	5. “CINV”[All Fields]
**Chemotherapy**	6. “drug therapy”[MeSH Terms] 7. “drug”[All Fields] AND “therapy”[All Fields] 8. “drug therapy”[All Fields] 9. “chemotherapy”[All Fields] 10. “antineoplastic agents”[MeSH Terms] 11. “antineoplastic”[All Fields] AND “agents”[All Fields] 12. “antineoplastic agents”[All Fields] 13. “antineoplastic”[All Fields] AND “agent”[All Fields] 14. “antineoplastic agent”[All Fields] 15. “antineoplastic agents”[Pharmacological Action] 16. OR (6-15)
**Nausea**	17. “nausea”[MeSH Terms] 18. “nausea”[All Fields] 19. OR (17, 18)
**Vomiting**	20. “vomiting”[MeSH Terms] 21. “vomiting”[All Fields] 22. OR (20, 21)
**Chemotherapy Induced Nausea and/or Vomiting (CINV)**	23. OR (19, 22) 24. AND (16, 23) 25. OR (5, 24)
**Risk Factors**	26. “risk factors”[MeSH Terms] 27. “risk”[All Fields] AND (“factors”[All Fields] OR “factor”[All Fields]) 28. “risk factors”[All Fields] 29. “risk factor”[All Fields] 30. OR (26-29)
**English Language**	31. English[Lang]
**Final Search Query**	32. AND (4, 25, 30, 31)

### Inclusion and Exclusion Criteria

The inclusion criteria include: (1) studies that reported patient-related risk factors of CINV, (2) any kind of clinical studies including prospective, retrospective, clinical trial, cross-sectional, cross-over, and case-control studies, (3) studies that do not provide any concomitant cancer treatment such as radiotherapy or surgery, and (4) adult patient population. We excluded any articles not written in English.

### Study Selection and Data Extraction

The study selection was performed in two steps. In the first step, we read the titles and abstracts of the citations by the search query to screen the articles based on the inclusion/exclusion criteria. In the second step, we read the full text of the citations selected by the first step. The search criteria did not limit by publication date; the earliest eligible article was published in 1989.

We abstracted information from the eligible full-text articles. **Table 2** presents the extracted information that includes the authors and year, study type, number of patients, type of cancer, emetic risk of the chemotherapy, and the factors that were identified as the predictors of CINV.

### Data Analysis

We extracted odds ratios and lower and upper value of 95% CI from each study if reported. Otherwise, an odds ratio was computed from the derived results. In order to provide a quantitative overview of the effect size of each risk factor, we performed meta-analysis of odds ratios applying the random effect model using the “meta” package in R (www.r-project.org). DerSimonian-Laird estimate (DerSimonian and Laird, 1986) was used for the random effects model. We created forest plots using the forest function in the “meta” package in R.

## Results

**Figure 1:**
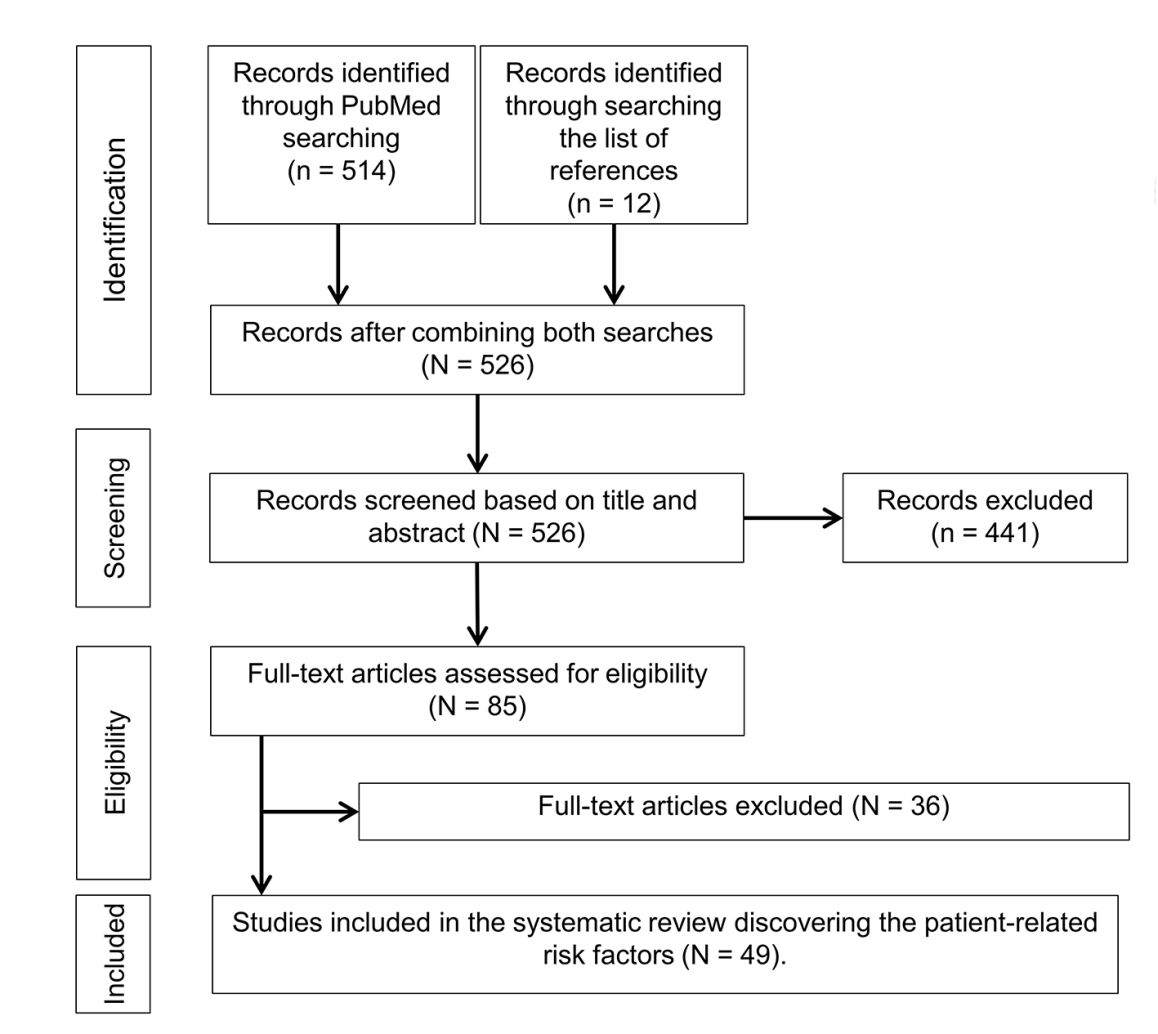
Trial flow diagram.

**Figure 1** presents the trial flow diagram used to identify the eligible articles for this study. A total of 514 articles were identified through the searches conducted in PubMed. We identified an additional 12 articles by reviewing the list of references. The titles and abstracts of those 526 articles were then screened for eligibility. A total of 441 articles were excluded based on title and abstract review because they did not meet the inclusion criteria described in the methods section. The remaining 85 articles were assessed in full-text for eligibility and data extraction. We excluded a total of 36 articles after full-text review. Finally, we included 49 articles in this study to elucidate the patient-related risk factors (n=49).

**Table 2** presents the patient-related risk factors from the 49 studies. Among those 49 studies, 18 studies were multicenter study and 31 studies were single-center study. The study types include retrospective (n = 6), prospective (n = 29), clinical trial (n = 11), cross-sectional (n = 1), cross-over (n = 1), and case-control (n = 1). The studies were performed in various countries around the world: USA (n = 6), Canada (n = 4), Singapore (n = 3), Italy (n = 3), Germany (n = 1), Japan (n = 18), Korea (n = 3), Malaysia (n = 1), Netherlands (n = 1), Sweden (n = 1), Taiwan (n = 1), UK (n = 1); four studies were performed in multiple countries (n = 6). A total of 21,569 patient-records were analyzed in the 49 studies. Fifteen studies focused on any cancer including both solid and hematological malignancies (n = 15), eleven studies focused on solid cancers only (n = 11), and 23 studies focused on a specific type of cancer: breast cancer (n = 10), colorectal cancer (n = 3), gastrointestinal cancer (n = 3), lung cancer (n = 1), gynecologic cancer (n = 3), and ovarian cancer (n = 1). Most of the research studied high and moderate emetic risk of the chemotherapies: high emetic risk only (n = 14), moderate emetic risk only (n = 11), low emetic risk only (n = 2), both high and moderate risk chemotherapies (n = 18), both moderate and low risk chemotherapies (n = 1), and high, moderate, and low risk chemotherapies (n = 3).

**Table 2:** Patient-related risk factors of Chemotherapy-Induced Nausea and Vomiting (CINV).

Author (Year)	# Patients	Cancer types	Emetic risk	Identified risk factors
(Shimokawa et al., 2019)	182	Gynecologic cancer	moderate	age
(Tsuji et al., 2019)	825	Solid	high	age, female sex
(Hayashi et al., 2018)	210	Any cancer	low	history of CINV, performance status
(Kawazoe et al., 2018)	103	Breast	high	Age <=55, BMI, alcohol intake
(Nawa-Nishigaki et al., 2018)	73	Breast	High	Age <55
(Hayashi et al., 2017)	222	Any cancer	low	history of nausea/vomiting
(Fujii et al., 2017)	186	Gastrointestinal	High	Female sex
(Uchida et al., 2017)	74	Lymphoma	High	Female sex, age <60, performance status, alcohol intake
(Dranitsaris et al., 2017)	1,198	Any cancer	High, moderate and low	Age <60, expectancy of CINV, number of hours of sleep the night before the chemotherapy, history of morning sickness, prior CINV, first cycle of chemotherapy
(Tsuji et al., 2017)	190	Colorectal	moderate	history of motion sickness
(Lee et al., 2017)	134	Breast	High and moderate	history of nausea/vomiting, chronotypes
(Takemoto et al., 2017)	370	Colorectal	Moderate	Female sex
(Di Mattei et al., 2016)	94	Gynecologic cancer	High, moderate, and low	age, alcohol intake, working status, history of CINV, state anxiety
(Baba et al., 2016)	192	Gastrointestinal	High and moderate	Female sex
(Rha et al., 2016)	332	Solid	High and moderate	Age <55, low alcohol intake, prior CINV, expectancy of CINV
(Hu et al., 2016)	898	Any cancer	High and moderate	Female sex
(Mizuno et al., 2016)	214	Gynecologic cancer	High and moderate	history of morning sickness, younger age
(Iihara et al., 2016)	779	Any cancer	High and moderate	female sex, Age < 60
(Molassiotis et al., 2016)	991	Any cancer	High and moderate	history of CINV, younger age, anxiety, expectancy of CINV
(Kitazaki et al., 2015)	133	Lung	High and moderate	None
(Tamura et al., 2015)	1,910	Any Cancer	High and moderate	Younger age, female sex, history of motion sickness, history of pregnancy-related nausea/vomiting, history of morning sickness and # of drinks per week < 5
(Molassiotis et al., 2014)	991	Solid	High and moderate	Age < 50, nausea before chemotherapy, prior CINV, history of CINV, anxiety, female sex
(Murakami et al., 2014)	92	Any cancer	Moderate	Age < 67, female sex
(Furukawa et al., 2014)	72	Gynecologic cancer	Moderate	Comorbidity (hypertension), history of pregnancy-related nausea/vomiting
(Celio et al., 2013)	405	Breast	Moderate	None
(Molassiotis et al., 2013)	336	Solid	High, moderate and low	Younger age, history of nausea/vomiting, trait anxiety, pain, first cycle of chemotherapy
(Sekine et al., 2013)	1,549	Any cancer	High and moderate	Female sex, age < 55 years, poor performance status, low alcohol intake (nonhabitual drinker) and nonsmoking habit
(Chan et al., 2012)	156	Gastrointestinal	Moderate	Prior CINV and moderate to severe anxiety
(Fleishman et al., 2012)	41	Colorectal	Moderate	Female sex and prior CINV
(Yap et al., 2012)	710	Solid	High and moderate	Fear of dying, fear of the worst, unable to relax, hot/cold sweats, nervousness, faintness, numbness
(Higa et al., 2012)	25	Solid	Moderate	Pretreatment ratio of substance-p and 5-HIAA/creatinine > 70
(Celio et al., 2012)	324	Solid	Moderate	Age < 50
(Hilarius et al., 2012)	277	Any cancer	High and moderate	Age < 65, female sex, # of drinks per week < 5
(Bourdeanu et al., 2012)	358	Breast	High	Asian race, private insurance, age ≤ 50 and GERD
(Warr et al., 2011)	866	Breast	Moderate	Age < 55, # of drinks per week < 5, history of morning sickness
(Hassan and Yusoff, 2010)	158	Breast	High	Race
(Hesketh et al., 2010)	1,043	Solid	High	Female sex, age <65, # of drinks per week < 5
(Roscoe et al., 2010)	1,696	Any cancer	High and moderate	Breast cancer, age < 40, expectancies and perceived susceptibility to nausea
(Shih et al., 2009)	91	Breast	High	Anxiety, prior CINV
(Dranitsaris et al., 2009)	200	Any cancer	High and moderate	Age < 40, gynecologic or genitourinary cancer, cancer stage I/II, no existing comorbidity, # of drinks per week < 7, chemotherapy cycle no. < 3, nonprescription drugs for emesis control before chemotherapy
(Petrella et al., 2009)	200	Any cancer	High and moderate	Age < 40, prior CINV, nausea/vomiting before chemotherapy, history of morning sickness, nonprescription drugs for emesis control before chemotherapy, chemotherapy cycle no. < 3, lower number of hours slept before the night of chemotherapy
(Colagiuri et al., 2008)	691	Any cancer	High	Expectancy of CINV
(Booth et al., 2007)	143	Breast	Moderate and low	Age < 40, no existing comorbidity, recent surgery, expectancy of CINV, # of drinks per week < 7, no food before chemotherapy, history of morning sickness
(Roscoe et al., 2004)	201	Breast	High and moderate	Expectancy of CINV
(Liaw et al., 2003)	400	Solid	High	Female sex
(Osoba et al., 1997)	832	Any cancer	High and moderate	Social functioning < 70, prechemotherapy nausea, female sex, # of drinks per week < 10
(Hursti et al., 1996)	101	Ovarian	High	Tumor burden ≥ 2cm, age ≥ 55
(du Bois et al., 1992)	92	Solid	High	Female sex, recurrent cancer
(Pollera and Giannarelli, 1989)	209	Solid	High	Age ≤ 55, female sex and poor performance status

We present the patient-related factors in two categories: the factors that were identified as the risk factors of CINV and the factors that were included in the data analysis and found to be insignificant.

### CINV Risk Factors

#### Age

**Figure 2:**
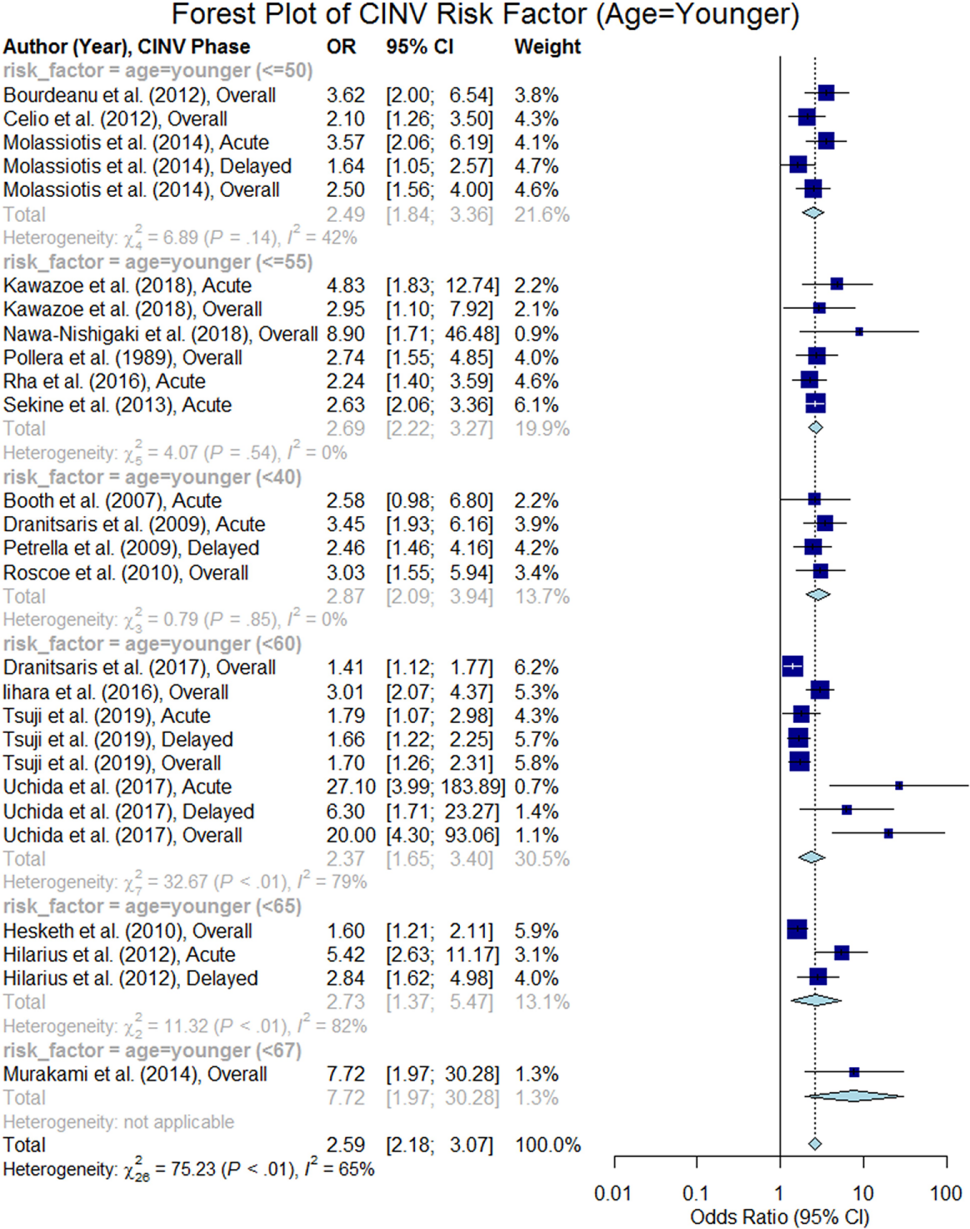
Forest plot of Chemotherapy-Induced Nausea and Vomiting (CINV) risk factor (age = younger).

**Figure 3:**
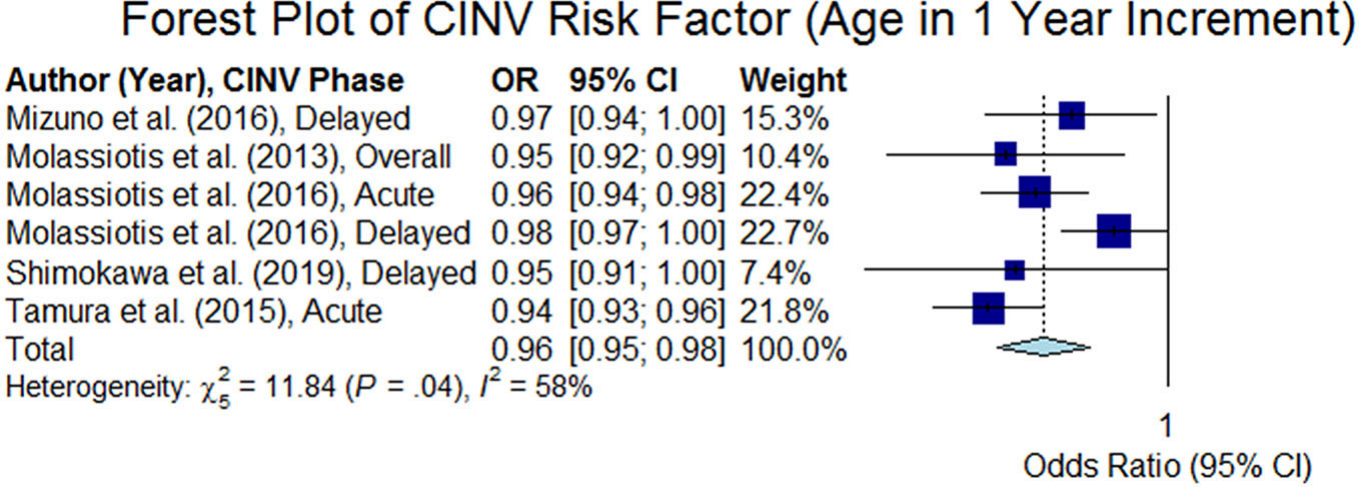
Forest plot of Chemotherapy-Induced Nausea and Vomiting (CINV) risk factor (age in 1 year increment).

Age was analyzed in 44 studies among which 27 studies (Pollera and Giannarelli, 1989; Hursti et al., 1996; Booth et al., 2007; Dranitsaris et al., 2009; Petrella et al., 2009; Hesketh et al., 2010; Roscoe et al., 2010; Warr et al., 2011; Bourdeanu et al., 2012; Celio et al., 2012; Hilarius et al., 2012; Molassiotis et al., 2013; Sekine et al., 2013; Molassiotis et al., 2014; Murakami et al., 2014; Tamura et al., 2015; Di Mattei et al., 2016; Iihara et al., 2016; Mizuno et al., 2016; Molassiotis et al., 2016; Rha et al., 2016; Dranitsaris et al., 2017; Uchida et al., 2017; Kawazoe et al., 2018; Nawa-Nishigaki et al., 2018; Shimokawa et al., 2019; Tsuji et al., 2019) confirmed that age is a risk factor of CINV. Out of those 27 studies, 26 studies confirmed that younger patients are at higher risk of CINV than older patients (p < 0.05). However, the age threshold varied from 40 to 67 in 21 studies (Pollera and Giannarelli, 1989; Hursti et al., 1996; Booth et al., 2007; Dranitsaris et al., 2009; Petrella et al., 2009; Hesketh et al., 2010; Roscoe et al., 2010; Warr et al., 2011; Bourdeanu et al., 2012; Celio et al., 2012; Hilarius et al., 2012; Sekine et al., 2013; Jordan et al., 2014; Murakami et al., 2014; Iihara et al., 2016; Rha et al., 2016; Dranitsaris et al., 2017; Uchida et al., 2017; Kawazoe et al., 2018; Nawa-Nishigaki et al., 2018; Tsuji et al., 2019) and six studies (Molassiotis et al., 2013; Tamura et al., 2015; Di Mattei et al., 2016; Mizuno et al., 2016; Molassiotis et al., 2016; Shimokawa et al., 2019) considered age as a continuous variable in which five studies reported required results for computing the summary odds ratio. The thresholds were as follows: age < 40 (n = 4), age ≤ 50 (n = 3), age ≤ 55 (n = 7), age < 60 (n = 4), age < 65 (n = 2), and age < 67 (n = 1) (see **Table 2**). Out of the 21 studies that analyzed the age risk factor using a threshold, we were able to extract odds ratio for 19 studies. The overall odds ratio was estimated to be 2.59 (95% CI 2.18–3.07); **Figure 2** presents a forest plot of those 19 studies. The forest plot of the five studies that regarded age as a continuous variable (1 year increment) is shown in **Figure 3**, and the summary odds ratio was estimated to be 0.96 (95% CI 0.95–0.98). This means that the risk of CINV is reduced by 4% with the increase of age by 1 year.

#### Sex

**Figure 4:**
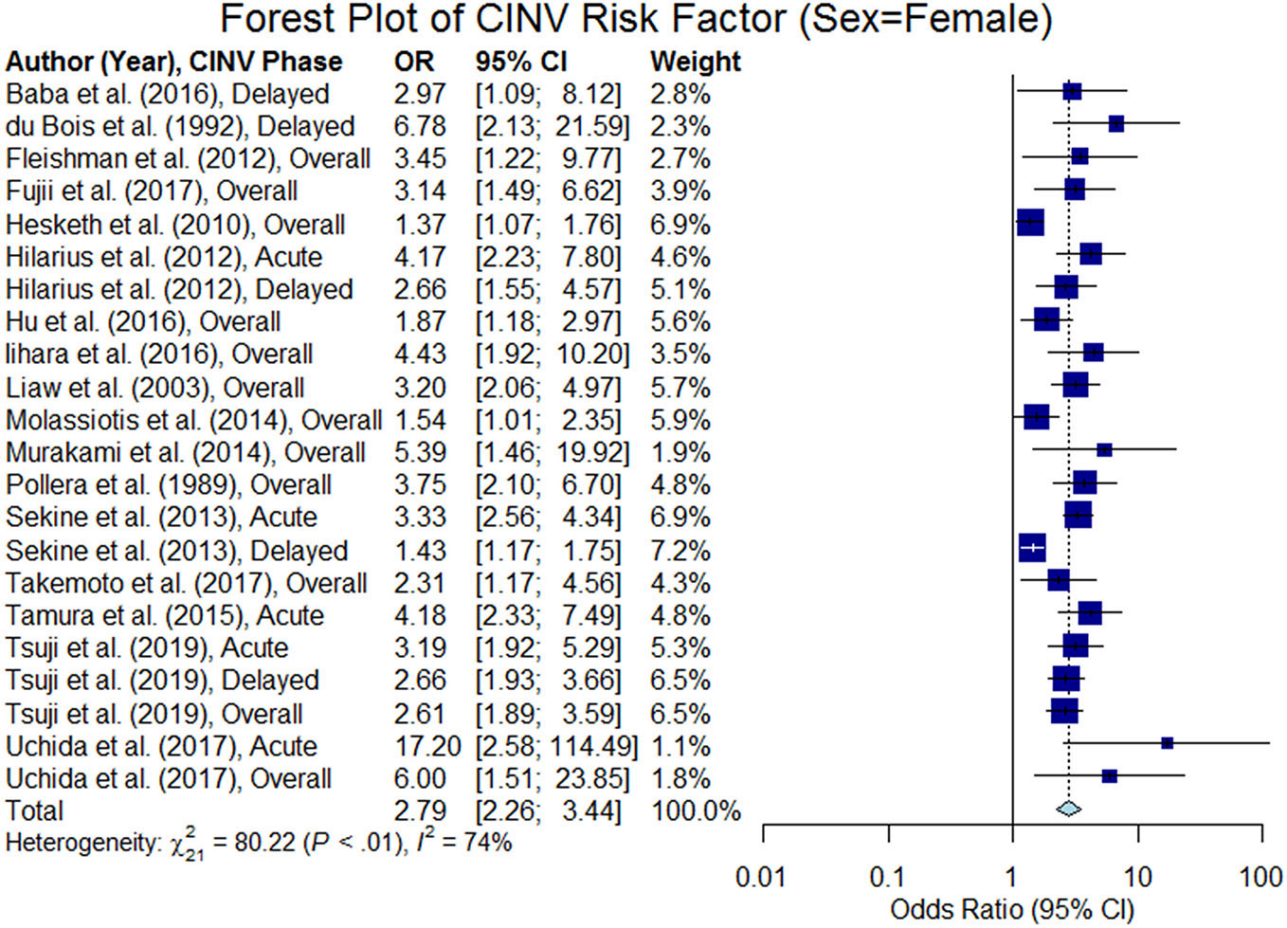
Forest plot of Chemotherapy-Induced Nausea and Vomiting (CINV) risk factor (sex = female).

Sex was analyzed in 32 studies and of those, 18 studies confirmed that female patients are at higher risk of CINV than male patients (p < 0.05). We were able to extract odds ratio from 17 studies (see the forest plot in **Figure 4**), and the summary odds ratio was estimated to be 2.79 (95% CI 2.26–3.44).

#### Alcohol Intake

**Figure 5:**
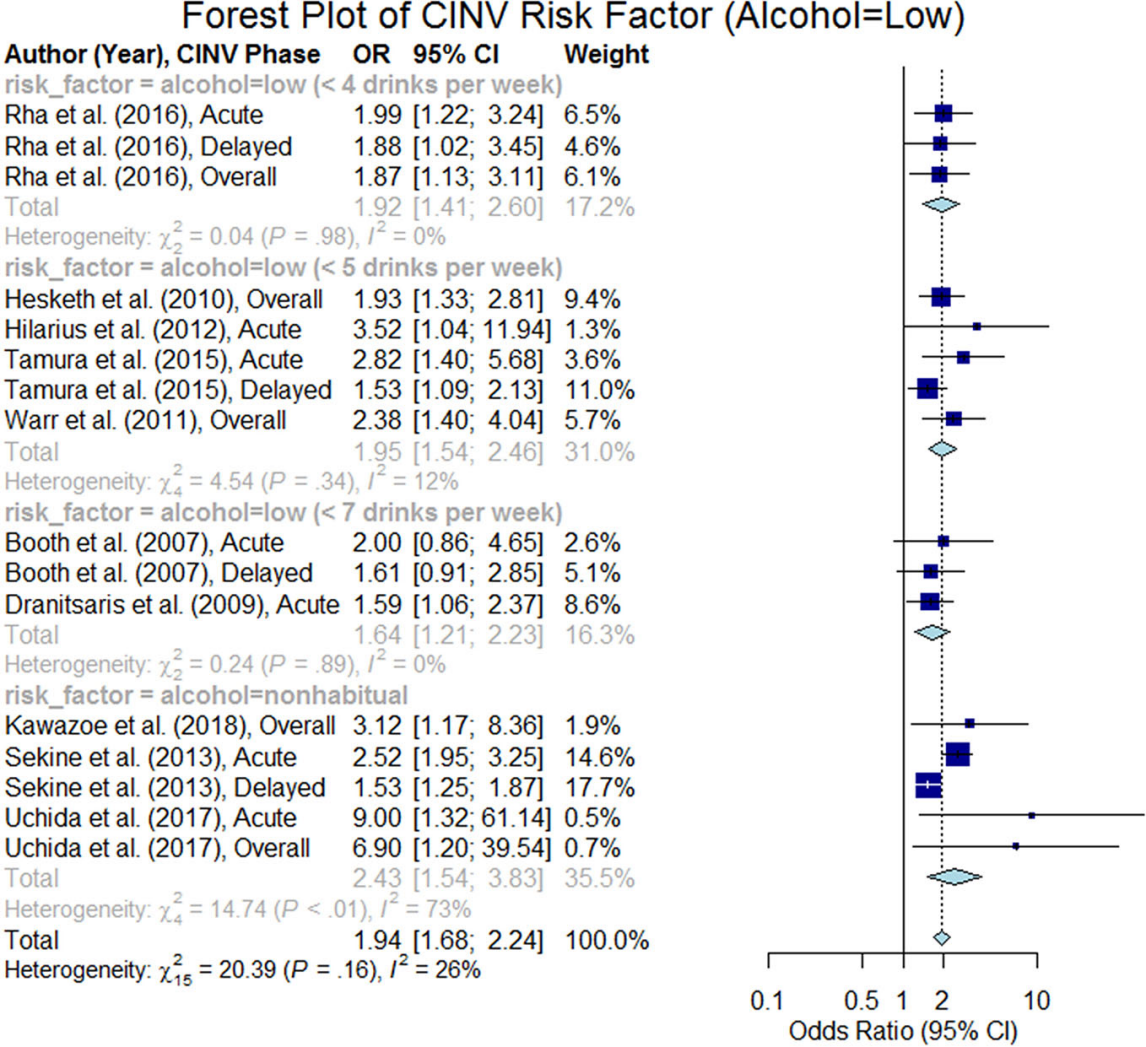
Forest plot of Chemotherapy-Induced Nausea and Vomiting (CINV) risk factor (alcohol = low).

The impact of chronic alcohol consumption habits on CINV was studied in 32 studies, in which 12 studies confirmed that patients with lower alcohol intake are at higher risk of CINV (p < 0.05). Eight studies defined low alcohol intake on the basis of the number of standard drinks per week; the thresholds were < 4 drinks (Rha et al., 2016) (n = 1), < 5 drinks (Hesketh et al., 2010; Warr et al., 2011; Hilarius et al., 2012; Tamura et al., 2015) (n = 4), < 7 drinks (Booth et al., 2007; Dranitsaris et al., 2009) (n = 2), and <10 drinks (Osoba et al., 1997) (n = 1). Four studies defined low alcohol intake as nonhabitual drinker (Sekine et al., 2013; Di Mattei et al., 2016; Uchida et al., 2017; Kawazoe et al., 2018). We were able to extract odds ratio from 10 studies out of 12 studies. The forest plot of these 10 studies (Booth et al., 2007; Dranitsaris et al., 2009; Hesketh et al., 2010; Warr et al., 2011; Hilarius et al., 2012; Sekine et al., 2013; Tamura et al., 2015; Rha et al., 2016; Uchida et al., 2017; Kawazoe et al., 2018) are presented in **Figure 5**, and the summary odds ratio was estimated to be 1.94 (95% CI 1.68–2.24). It is important to acknowledge that the studies may have inherent biases due to the limitations of measuring alcohol use.

#### History of Nausea/Vomiting

**Figure 6:**
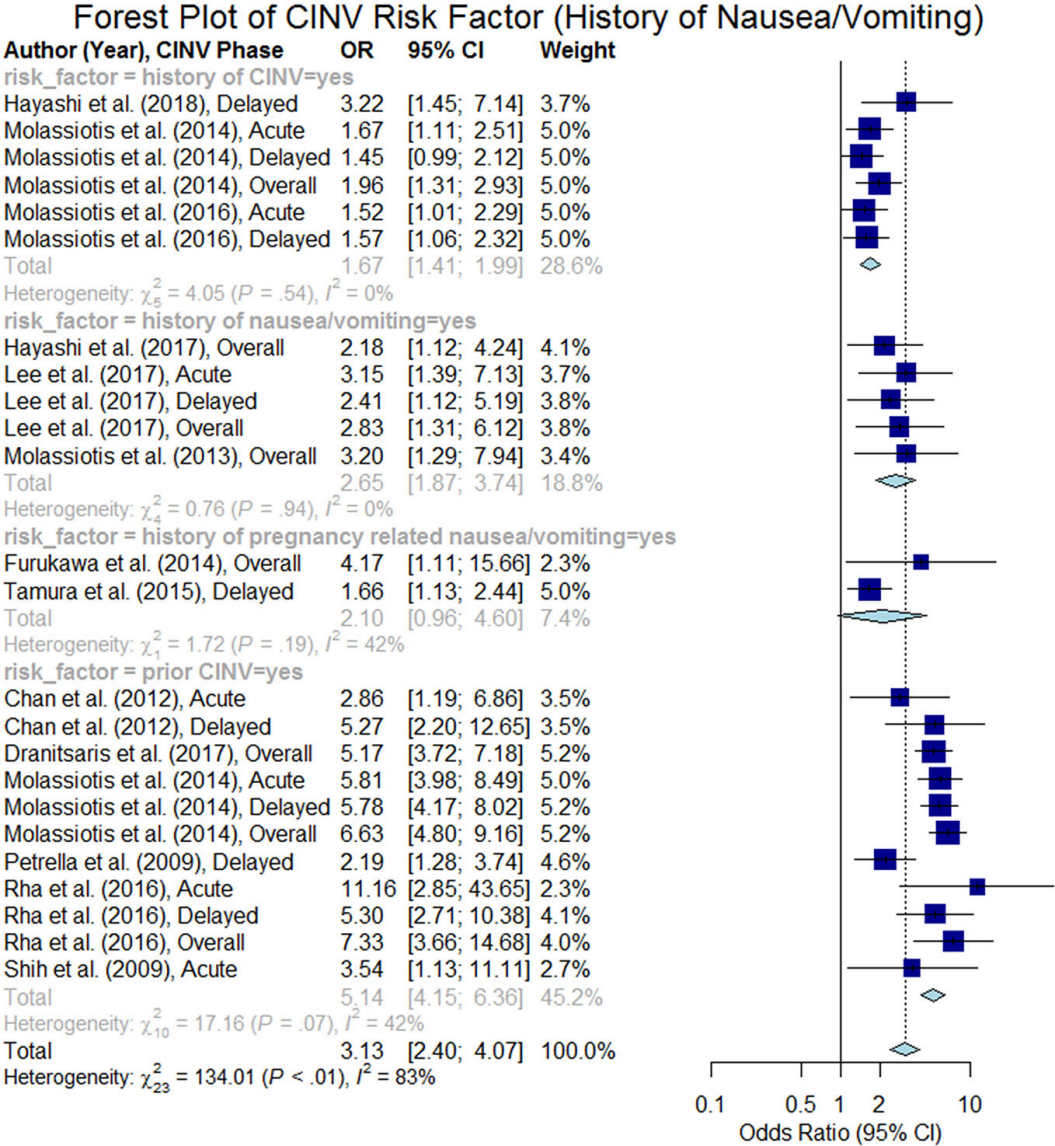
Forest plot of Chemotherapy-Induced Nausea and Vomiting (CINV) risk factor (history of CINV and/or pregnancy-related nausea/vomiting).

A total of 25 studies analyzed the history of nausea/vomiting including history of nausea/vomiting (unspecified), history of CINV, prior CINV, and history of pregnancy related nausea/vomiting. Six studies reported that patients experiencing CINV during the prior chemotherapy treatment cycle are at higher risk of CINV during their current cycle (summary odds ratio 5.14, 95% CI 4.15–6.36). Also, four studies reported that the patients with history of CINV at an earlier treatment were at higher risk of CINV (summary odds ratio 1.67, 95% CI 1.41–1.99). Two studies found that female patients experiencing severe nausea/vomiting during their prior pregnancies are at higher risk of CINV (summary odds ratio 2.10, 95% CI 0.96–4.60). Three studies reported history of nausea/vomiting as unspecified and found significant impact on CINV (summary odds ratio 2.65, 95% CI 1.87–3.74). The summary odds ratio for all of the studies was estimated to be 3.13 (95% CI 2.40–4.07) as shown in **Figure 6**.

#### History of Morning Sickness

**Figure 7:**
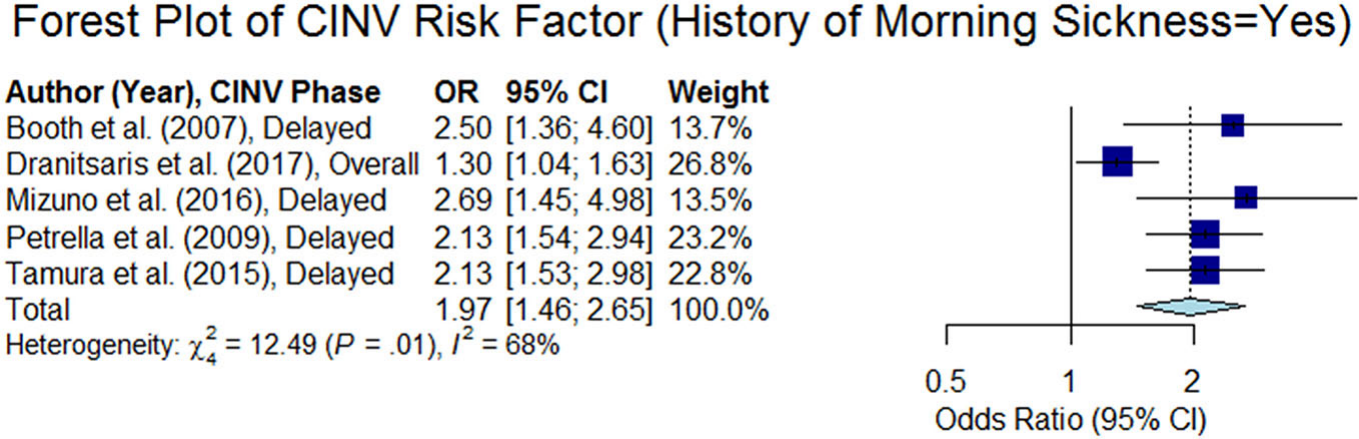
Forest plot of Chemotherapy-Induced Nausea and Vomiting (CINV) risk factor (history of morning sickness = yes).

Six studies (out of 15 studies that analyzed this variable) found that patients who experienced morning sickness were at higher risk of CINV (Booth et al., 2007; Petrella et al., 2009; Warr et al., 2011; Tamura et al., 2015; Mizuno et al., 2016; Dranitsaris et al., 2017). We computed the odds ratio from five studies and the forest plot of those studies are presented in **Figure 7**. The overall summary odds ratio was estimated to be 1.97 (95% CI 1.46–2.65, p < 0.05).

#### Anxiety

**Figure 8:**
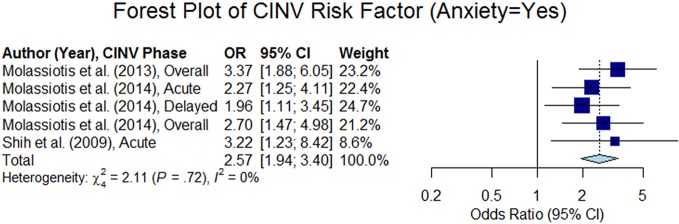
Forest plot of Chemotherapy-Induced Nausea and Vomiting (CINV) risk factor (anxiety = yes).

Moderate to high anxiety was found to be a risk factor of CINV in six studies (p < 0.05), out of 12 studies that analyzed that variable. Most of the studies determined the level of anxiety using a Likert scale (e.g., a four-point Likert scale is graded as none, mild, moderate, and high) or a 0–100-mm visual analog scale (VAS). We computed odds ratio from four studies. Three studies out of the four studies considered anxiety as a categorical variable (anxiety = yes or, no) and estimated summary odds ratio was 2.57 (95% CI 1.94–3.40) as shown in **Figure 8**. Molassiotis et al. (2016) considered anxiety as a continuous variable (0–100-mm VAS) and reported overall odds ratio to be 1.01 (95% CI 1.01–1.01). Yap et al. (2012) used the Beck Anxiety Inventory (BAI) to capture the state of anxiety using 21 anxiety-related symptoms. This study reported that seven anxiety-related symptoms were strongly related to the CINV (p < 0.05): fear of dying, fear of the worst, unable to relax, hot/cold sweats, nervousness, faintness, and numbness.

#### Expectancy of CINV

**Figure 9:**
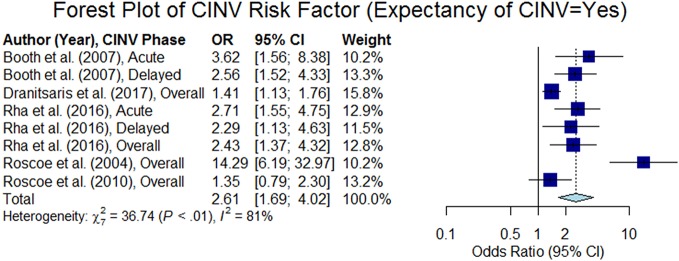
Forest plot of Chemotherapy-Induced Nausea and Vomiting (CINV) risk factor (expectancy of cinv = yes).

Patients who expect nausea/vomiting after their chemotherapy, regardless of previous experience, are more likely to experience CINV. This attribute was analyzed in 10 studies, out of which seven studies confirmed the hypothesis (p < 0.05). Odds ratios were extracted from six studies. Five studies out of the six studies treated this variable as categorical (yes or no) and estimated summary odds ratio was 2.61 (95% CI 1.69–4.02), as shown in **Figure 9**. Molassiotis et al. (2016) considered expectancy of nausea as a continuous variable (0–100-mm VAS) and reported the odds ratio to be 1.012 (95% CI 1.006–1.018) in acute phase and 1.011 (95% CI 1.005–1.016) in delayed phase.

#### Chemotherapy Cycle Number

This variable was analyzed in seven studies, of which, two studies (Dranitsaris et al., 2009; Petrella et al., 2009) demonstrated that patients in earlier cycles (cycle no. < 3) are at higher risk of CINV. A similar result was reported by Molassiotis et al. (2013) and Dranitsaris et al. (2017) that patients in the first-cycle of chemotherapy are at higher risk of CINV. However, Shih et al. (2009), Hayashi et al. (2017) and Di Mattei et al. (2016) found no relation of chemotherapy cycle numbers to the risk of CINV.

#### Comorbidities

**Figure 10:**
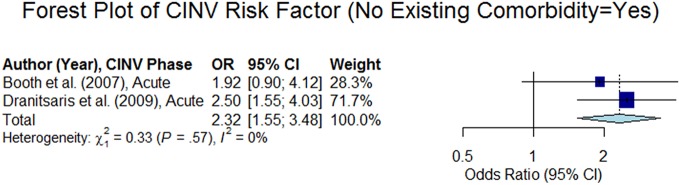
Forest plot of Chemotherapy-Induced Nausea and Vomiting (CINV) risk factor (no existing comorbidity = yes).

Two out of seven studies showed that existing comorbidities (such as diabetes, cardiovascular, gastrointestinal, musculoskeletal, thyroid, and other diseases) reduce the risk of CINV (Booth et al., 2007; Dranitsaris et al., 2009) (summary odds ratio: 2.32, 95% CI 1.55–3.48, p < 0.05, see the forest plot in **Figure 10**). This is consistent with the result as reported in Furukawa et al. (2014), which demonstrated that hypertension medication reduces the risk of CINV in the overall phase (odds ratio: 0.096, 95% CI 0.016–0.585, p < 0.05). In contrast, Bourdeanu et al. (2012) reported that Gastroesophageal Reflux Disease (GERD) increases the risk of CINV (odds ratio: 3.32, 95% CI 1.15–9.31, p < 0.05) and Lee et al. (2017) reported that late chronotype increases the risk of CINV (odds ratios: acute phase = 3.62, 95% CI 1.33–9.86, p < 0.05; delayed phase = 3.3, 95% CI 1.24–8.77, p < 0.05, overall phase = 3.53, 95% CI 1.27–9.79, p < 0.05).

#### Cancer Type

This risk factor was analyzed in 15 studies. Only two studies found that specific cancers have effect on the risk of CINV: breast cancer increased the risk (overall phase odds ratio: 1.59, 95% CI 1.1–2.38, p < 0.05) (Roscoe et al., 2010) and genitourinary or gynecologic cancer reduced the risk in the acute phase (odds ratio: 0.49, 95% CI 0.3–0.8, p < 0.05) (Dranitsaris et al., 2009). Because almost breast cancer patients are female and female patients are at higher risk of CINV, there might be a collinearity between these two factors.

#### Performance Status (PS)

**Figure 11:**
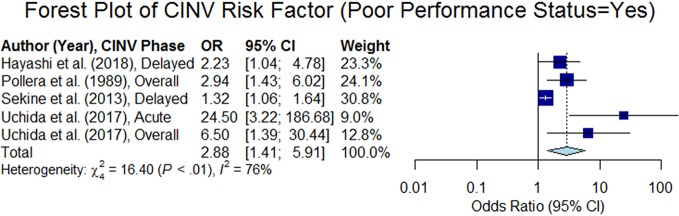
Forest plot of Chemotherapy-Induced Nausea and Vomiting (CINV) risk factor (poor performance status = yes).

PS, as estimated by the Eastern Cooperative Oncology Group (ECOG) PS scale, is graded at six levels from 0 to 5, where a value greater than one indicates poor PS (i.e., a patient is not fully active). This variable was analyzed in nine studies, out of which four studies reported that poor PS is a risk factor of CINV (Pollera and Giannarelli, 1989; Sekine et al., 2013; Uchida et al., 2017; Hayashi et al., 2018). The overall odds ratio was estimated to be 2.88 (95% CI 1.41–5.91, p < 0.05, see the forest plot in **Figure 11**).

#### Race

Five studies analyzed the impact of race on CINV, of which two studies demonstrated that patients from certain races are at higher risk of CINV. Bourdeanu et al. (2012) reported that Asian female breast cancer patients are at higher risk of CINV than Caucasian, African American, and Hispanic breast cancer patients (the overall phase odds ratio was 2.12 and 95% CI was 1.18–3.81, p < 0.05). Hassan and Yusoff (2010) studied the three races in Malaysia (Malay, Chinese, and Asian Indians) and found that the Chinese patients were at higher risk of CINV in comparison to the other two races.

#### Use of Nonprescription Drugs

Two studies demonstrated that the use of nonprescription drugs at home before chemotherapy, for nausea and vomiting control, significantly increased the risk of CINV (Dranitsaris et al., 2009; Petrella et al., 2009). Both studies analyzed the same dataset separately for the acute and delayed phase of CINV. The names of the nonprescription drugs were not reported in those studies. The odds ratios were 2.49 for the acute phase (95% CI 1.4–4.43, p < 0.05) (Dranitsaris et al., 2009) and 11.93 (95% CI 5.26–26.8, p < 0.05) (Petrella et al., 2009) for the delayed phase.

#### History of Motion Sickness

Three out of 25 studies reported that the history of motion sickness is a risk factor of CINV (Shih et al., 2009; Tamura et al., 2015; Tsuji et al., 2017). Motion sickness is defined as sickness that is caused due to motion while traveling by car, train, airplanes, or boats. Tamura et al. (2015) reported the odds ratio to be 2.183 (95% CI 1.292–3.689, p < 0.05) for acute phase and Tsuji et al. (2017) reported that the odds ratio to be 3.89 (95% CI 1.49–10.19, p < 0.05) for delayed phase.

#### Cancer Stage

There are five stages in cancer: stage 0, I, II, III, and IV. The variable “cancer stage” was analyzed in eight studies but only one study reported that patients with early stages of cancer (stages I and II) are at higher risk of CINV during the acute phase (odds ratio: 1.68, 95% CI 1.08–2.62, p < 0.05) (Dranitsaris et al., 2009); the reasoning behind this finding was not discussed in this study. In contrast, cancer stage was reported to be insignificant for developing CINV during the delayed phase using the same dataset (Petrella et al., 2009).

#### Sleeping Before Chemotherapy

Petrella et al. (2009) reported that with decreased hours of sleep in the night before chemotherapy, the probability of CINV during delayed phase increased (odds ratio: 1.163, 95% CI 1.053–1.299, p < 0.05). Similar results was reported by Dranitsaris et al. (2017) for overall phase (odds ratio: 1.34, 95% CI 1.1–1.48, p < 0.05). In contrast, this variable had no significant impact on developing CINV during acute phase using the same dataset (Dranitsaris et al., 2013). There are two other studies that analyzed the variable, but found no impact on developing CINV.

#### Meal Before Chemotherapy

Booth et al. (2007) reported that having no food before chemotherapy increased the probability of CINV in the acute phase (odds ratio: 6.81, 95% CI 2.5, 18.6, p < 0.05). As such, this study suggested that the importance of eating a small amount of food prior to treatment should be emphasized during prechemotherapy education so that the risk of developing CINV could be reduced. Three other studies found no impact on CINV.

#### Recent Surgery

Booth et al. (2007) reported that having surgery within the past three months of chemotherapy increased the probability of CINV in the acute phase (odds ratio: 3.8, 95% CI 1.35–11, p < 0.05). Two other studies found no impact on CINV.

#### Smoking History

Four studies conducted in Japan by Sekine et al. (2013), Furukawa et al. (2014), Kawazoe et al. (2018), and Nawa-Nishigaki et al. (2018) analyzed the impact of smoking on CINV. Only Sekine and colleagues Sekine et al. (2013) reported that nonsmokers are at higher risk of CINV than smokers in the acute phase (odds ratio: 2.02, 95% CI 1.54–2.64, p < 0.05).

#### Neurotransmitter Measurements

Substance-P and 5-HIAA/creatinine are two neurotransmitter measurements that are measured from blood and urine sample respectively. Higa et al. (2012) studied the correlation of these neurotransmitters with the development of CINV due to moderately emetogenic chemotherapy (MEC). This study reported that the ratio of substance-P to 5-HIAA/creatinine greater than 70 is significantly associated with the development of CINV during delayed phase due to MEC (odds ratio: 34.667, 95% CI 3.056–393.203, p < 0.05).

#### Private/Public Insurance

Bourdeanu et al. (2012) performed a study on breast cancer patients and demonstrated that patients having private health insurance are at higher risk of CINV than the ones having public health insurance such as state or federally funded insurances (odds ratio: 2.13, 95% CI 1.23–3.78, p < 0.05).

#### Social Functioning

Social functioning is a variable within the health-related quality-of-life (HQL) indicator. Osoba et al. (1997) reported that a social functioning score less than 70 is a significant predictor of CINV. The social functioning score is measured by the European Organization for Research and Treatment (EORTC) Core Quality of Life Questionnaire (QLQ-C30). The score ranges from 0 to 100, and a higher score indicates a higher level of functioning.

#### Nausea/Vomiting Before Chemotherapy

**Figure 12:**
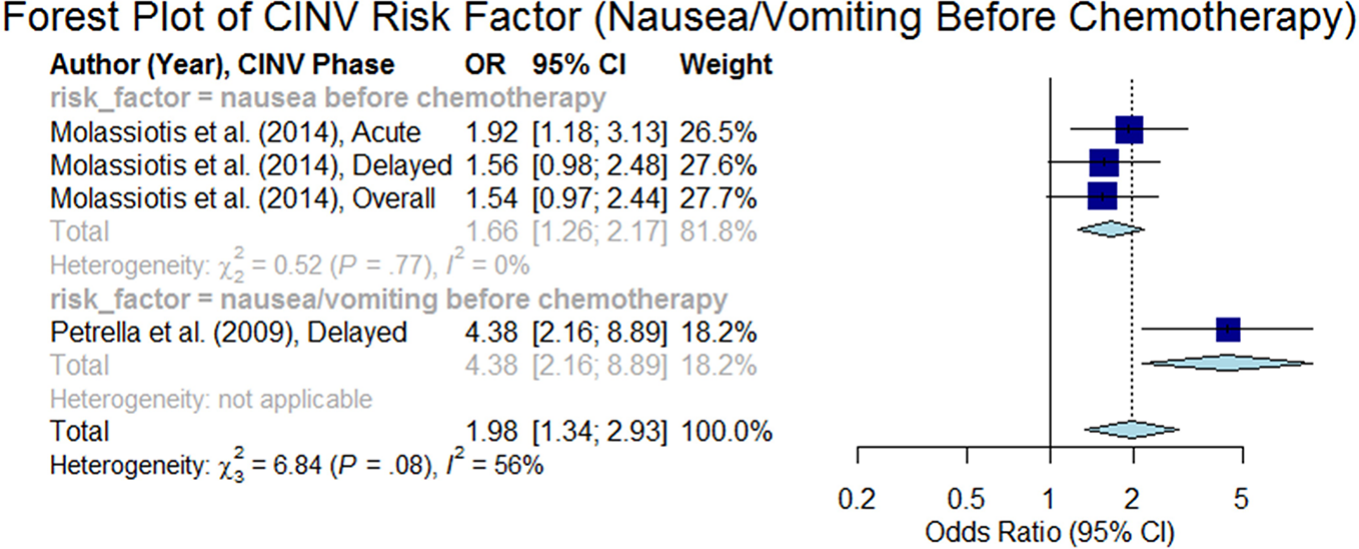
Forest plot of Chemotherapy-Induced Nausea and Vomiting (CINV) risk factor (nausea/vomiting before chemotherapy).

Petrella et al. (2009) demonstrated that acute nausea/vomiting before chemotherapy is a significant predictor of CINV during the delayed phase (odds ratio 4.38, 95% CI 2.16–8.90), but not in the acute phase (Dranitsaris et al., 2009). Both of these studies (Dranitsaris et al., 2009; Petrella et al., 2009) used the same dataset. Molassiotis et al. (2014) also supported that nausea prior to chemotherapy was a predictor of CINV, but this finding was significant for both the delayed and the acute phases. The overall summary odds ratio was estimated to be 1.98 (95% CI 1.34–2.93, p < 0.05) as presented in the forest plot in **Figure 12**.

#### Tumor Burden

Tumor burden/load is indicative of the amount of cancer in the body. Hursti et al. (1996) categorized the tumor burden into two groups: (1) minimal tumor burden was defined as the diameter of the greatest residual tumor being less than two centimeters, and (2) large tumor burden was defined as the diameter of the greatest residual tumor being greater or equal to two centimeters. This study reported that the ovarian cancer patients with larger tumor burdens are at higher risk of CINV.

#### Cancer Recurrence (New/Recurrent)

du Bois et al. (1992) demonstrated that recurrent cancer patients are at higher risk of CINV than new cancer patients.

#### Symptom Distress Score

Molassiotis et al. (2013) used Symptom Distress Score to measure the symptoms present before chemotherapy. This scale measures the symptom distress for a total of 13 items such as nausea, appetite, insomnia, pain, fatigue, bowel pattern, concentration, appearance, outlook, breathing, cough, frequency of nausea, and frequency of pain. Molassiotis et al. (2013) reported that out of these 13 items, only pain was linked with the development of CINV (overall phase odds ratio: 1.69, 95% CI 1.03–2.77, p < 0.05).

#### Body Mass Index (BMI)

BMI was analysed in five studies. Kawazoe et al. (2018) reported that BMI <27.5 kg/m^2^ are at higher risk of CINV (odds ratio: acute phase −4.19, 95% CI 1.15–15.26, p < 0.05; overall phase −3.94, 95% CI 1.11–13.98, p < 0.05).

#### Working Status

In a study with gynecologic cancer by Di Mattei et al. (2016), women who were involved in a working activity (both part-time and full-time) during chemotherapy treatments may experience less CINV (acute phase odds ratio 0.088, p=0.002 and delayed phase odds ratio 0.331, p=0.045) (Di Mattei et al., 2016).

### Nonsignificant Factors

A total of 17 patient-related factors were found to be insignificantly associated to the prediction of CINV in any study. These factors are history of previous chemo (n=6), depression, (n=2), education (n=2), marital status (n=1), religion (n=1), weight (n=1), frequency of chemotherapy cycle (n=1), number of risk factors (n=1), drowsiness (n=1), neuroticism (n=1), autonomic perception (n=1), menopause (n=1), defecations (bowel movement) (n=1), history of radiotherapy (n=1), opioid use (n=1), social jetlag (n=1), ascites (n=1), opioid use (n=1), and time since diagnosis (n=1).

## Discussion

A significant amount of research has been performed to identify patient-related risk factors of CINV. We have identified 49 studies through a systematic literature review. These studies analyzed a total of 47 patient-related variables. Among them, 28 factors were found to have a significant positive impact on the risk of CINV. Three factors are demographic-related: younger patients, female sex, and Asian race. A total of 17 factors are innate to patient's physiology or influenced by physiology, and may alter the pathophysiology of CINV (i.e., intrinsic); these factors include history of CINV or pregnancy-related nausea/vomiting, anxiety, expectancy of CINV, absence of comorbidities, cancer type (i.e., breast, genitourinary or gynecologic cancer), history of morning sickness, poor PS, history of motion sickness, early stage of cancer, lower number of hours of sleep before chemotherapy, recent surgery within the past three months, a higher ratio of neurotransmitter measurements, nausea/vomiting before chemotherapy, larger tumor burden, recurrent cancer, higher symptom distress score, and BMI. Seven factors may influence the pathophysiology of CINV and are external in nature (i.e., extrinsic). These factors include low alcohol intake, patients at earlier cycles (≤ 3) of chemotherapy, use of nonprescription drugs at home for emesis control before chemotherapy, no food intake before chemotherapy, nonsmoking habit, having private insurance, low social functioning, and working status.

None of the studies considered all the 47 variables together in their studies. The average number of variables included per study was 6.55 with a minimum of 1 and a maximum of 16 (median = 5 and standard deviation = 3.69). The average number of significant risk factors that were reported per study was 2.67 with a minimum of 0 and a maximum of 7 (median = 2 and standard deviation = 1.87). The maximum number of patient-related risk factors that were reported by a single study was 7 (n=3) (Booth et al., 2007; Dranitsaris et al., 2009; Petrella et al., 2009) followed by 6 (n=2) (Tamura et al., 2015; Dranitsaris et al., 2017) and 5 (n=4) (Molassiotis et al., 2013; Sekine et al., 2013; Molassiotis et al., 2014; Di Mattei et al., 2016).

**Table 3** presents the seven most important risk factors that were identified by at least five studies with notable summary odds ratios. These factors can be captured readily at the time of clinical encounters or assessment, and be used in the process of clinical decision making. It would be most useful if a validated clinical decision making tool such as risk scoring could be produced based on those risk factors and the emetogenicity of chemotherapies.

**Table 3:** Important risk factors of Chemotherapy-Induced Nausea and Vomiting (CINV).

Risk factors	Number of studies confirmed the risk factor	Summary odds ratios (p < 0.05)	95% CI
History of CINV and/or Pregnancy-related Nausea/Vomiting	15	3.13	2.40–4.07
Sex = Female	18	2.79	2.26–3.44
Expectancy of CINV = Yes	6	2.61	1.69–4.02
Age = Younger	27	2.59	2.18–3.07
Anxiety = Yes	6	2.57	1.94–3.40
History of Morning Sickness = Yes	6	1.97	1.46–2.65
Alcohol = Low	12	1.94	1.68–2.24

The significance of this study is threefold. First, the current CINV guidelines that are basically based on emetic risks of chemotherapies can be considerably augmented using the findings from this review study (i.e., a comprehensive list of significant patient-related factors) so that oncologists can consider these factors when they predict CINV. Second, the findings of the study can be used to discover associations among those patient-related factors. While it is important to identify individual patient-related factors, the more important research would be to discover how those individual factors are associated with one another in terms of the risk of CINV. Third, this review may lay the foundation for improved CINV prediction models. Currently, CINV prediction models based on antiemetics and patient-related factors are unsatisfactory (Dranitsaris et al., 2009; Petrella et al., 2009; Bouganim et al., 2012; Dranitsaris et al., 2013; Molassiotis et al., 2013). This indicates that associations among patient-related factors are not fully identified, and/or there are some important, yet hidden, patient-related factors.

The study has some limitations and challenges. We might miss a few relevant studies. Retrieving all relevant documents is technically infeasible for all literature review studies, which is a well-known factor in the information retrieval field. In order to collect as many relevant studies as possible, we adopted a “broad” search strategy at the cost of precision. The studies included in this systematic review had various methodological designs and various statistical measures. Other variations include different phases (acute, delayed or overall) of CINV, various cycles of chemotherapy. One of the major challenges in this study was that all articles do not include the results for all phases (acute, delayed and overall) of CINV. As a result, in order to synthesize the analysis results of studies with different designs into one summary effect size for the entire phase, we used all the reported odds ratios for each phase and applied the random effect model instead of the fixed effect model. We excluded the pharmacogenomics studies. Pharmacogenomics was not within the scope of this study because the pharmacogenomics studies of CINV are sparse and limited. A review conducted by Sugino and Janicki (2015) on the pharmacogenomics of CINV summarized that “the role of pharmacogenetics in mechanisms of CINV has not been fully unravelled, and it is premature to implement results of pharmacogenetic association studies of antiemetic drugs in clinical practice.” With the improvement in pharmacogenomics studies on CINV, a future systematic review will be needed to summarize the outcomes.

## Conclusion

This systematic review study has identified and summarized patient-related factors scattered over various studies that significantly impact the risk of CINV. The identification of patients at high risk for CINV based on key risk factors prior to the initiation of a chemotherapy regimen is imperative. Oncologists may be able to selectively focus on more comprehensive antiemetic treatments on high risk patients. Although there are 28 significant risk factors, most studies used only two or three significant factors as well as many nonsignificant factors. Therefore, future studies are needed that include as many significant risk factors as possible to see how, in combination, they may affect the risk of CINV.

## Author Contributions

AM contributed in conceptualization, design, validation, formal analysis, investigation, data curation, writing the original draft, reviewing, editing, and project administration. AH contributed in conceptualization, methodology, investigation, reviewing, editing, and supervision. BL contributed in investigation, reviewing, and editing. IY contributed in conceptualization, design, reviewing, editing, supervision, and project administration.

## Conflict of Interest

The authors declare that the research was conducted in the absence of any commercial or financial relationships that could be construed as a potential conflict of interest.

## References

[B1] Adel N. (2017). Overview of chemotherapy-induced nausea and vomiting and evidence-based therapies. Am. J. Manage. Care 23, S259–S265. 28978206

[B2] Baba Y. Baba H. Yamamoto S. Shimada H. Shibata T. Miyazaki T. (2016). Chemotherapy-induced nausea and vomiting is less controlled at delayed phase in patients with esophageal cancer: a prospective registration study by the CINV Study Group of Japan. Dis. Esophagus. 30 (2), 1–7. 10.1111/dote.12482. n/a-n/a. 27001532

[B3] Booth C. M. Clemons M. Dranitsaris G. Joy A. Young S. Callaghan W. (2007). Chemotherapy-induced nausea and vomiting in breast cancer patients: a prospective observational study. J. Support. Oncol. 5, 374–380. 17944146

[B4] Bouganim N. Dranitsaris G. Hopkins S. Vandermeer L. Godbout L. Dent S. (2012). Prospective validation of risk prediction indexes for acute and delayed chemotherapy-induced nausea and vomiting. Curr. Oncol. 19, e414–e421. 10.3747/co.19.1074 23300365 PMC3503672

[B5] Bourdeanu L. Frankel P. Yu W. Hendrix G. Pal S. Badr L. (2012). Chemotherapy-induced nausea and vomiting in Asian women with breast cancer receiving anthracycline-based adjuvant chemotherapy. J. Support. Oncol. 10, 149–154. 10.1016/j.suponc.2011.10.007 22222249

[B6] Celio L. Denaro A. Agustoni F. Bajetta E. (2012). Palonosetron Plus 1-Day Dexamethasone for the Prevention of Nausea and Vomiting Due to Moderately Emetogenic Chemotherapy: Effect of Established Risk Factors on Treatment Outcome in a Phase III Trial. J. Support. Oncol. 10, 65–71. 10.1016/j.suponc.2011.06.007 22005217

[B7] Celio L. Bonizzoni E. Bajetta E. Sebastiani S. Perrone T. Aapro M. S. (2013). Palonosetron plus single-dose dexamethasone for the prevention of nausea and vomiting in women receiving anthracycline/cyclophosphamide-containing chemotherapy: Meta-analysis of individual patient data examining the effect of age on outcome in two phase I. Support. Care Cancer 21, 565–573. 10.1007/s00520-012-1558-9 22869054 PMC3538015

[B8] Chan A. Tan S. H. Low X. H. Yap K. Y.-L. (2012). Antiemetic effectiveness and nausea and vomiting incidence during capecitabine and oxaliplatin chemotherapy. Nurs. Res. 61, 405–412. 10.1097/NNR.0b013e3182691438 22960588

[B9] Cohen L. de Moor C. A. Eisenberg P. Ming E. E. Hu H. (2007). Chemotherapy-induced nausea and vomiting: incidence and impact on patient quality of life at community oncology settings. Support. Care Cancer 15, 497–503. 10.1007/s00520-006-0173-z 17103197

[B10] Colagiuri B. Roscoe J. A. Morrow G. R. Atkins J. N. Giguere J. K. Colman L. K. (2008). How do patient expectancies, quality of life, and postchemotherapy nausea interrelate? Cancer 113, 654–661. 10.1002/cncr.23594 18521919 PMC3079444

[B11] DerSimonian R. Laird N. (1986). Meta-analysis in clinical trials. Control. Clin. Trials 7, 177–188. 10.1016/0197-2456(86)90046-2 3802833

[B12] Di Mattei V. E. Carnelli L. Carrara L. Bernardi M. Crespi G. Rancoita P. M. V. (2016). Chemotherapy-Induced Nausea and Vomiting in Women With Gynecological Cancer: A Preliminary Single-Center Study Investigating Medical and Psychosocial Risk Factors. Cancer Nurs. 39, E52–E59. 10.1097/NCC.0000000000000342 26895414

[B13] Dranitsaris G. Joy A. Young S. Clemons M. Callaghan W. Petrella T. (2009). Identifying patients at high risk for nausea and vomiting after chemotherapy: the development of a practical prediction tool. I. Acute nausea and vomiting. J. Support Oncol. 7, W1–W8.

[B14] Dranitsaris G. Bouganim N. Milano C. Vandermeer L. Dent S. Wheatley-Price P. (2013). Prospective validation of a prediction tool for identifying patients at high risk for chemotherapy-induced nausea and vomiting. J. Support. Oncol. 11, 14–21. 10.1016/j.suponc.2012.05.001 22763232

[B15] Dranitsaris G. Molassiotis A. Clemons M. Roeland E. Schwartzberg L. Dielenseger P. (2017). The development of a prediction tool to identify cancer patients at high risk for chemotherapy-induced nausea and vomiting. Ann. Oncol. Off. J. Eur. Soc Med. Oncol. 28, 1260–1267. 10.1093/annonc/mdx100 PMC5452068 28398530

[B16] du Bois A. Meerpohl H. G. Vach W. Kommoss F. G. Fenzl E. Pfleiderer A. (1992). Course, patterns, and risk-factors for chemotherapy-induced emesis in cisplatin-pretreated patients: a study with ondansetron. Eur. J. Cancer 28, 450–457. 10.1016/S0959-8049(05)80075-9 1534250

[B17] Fleishman S. B. Mahajan D. Rosenwald V. Nugent A. V. Mirzoyev T. (2012). Prevalence of Delayed Nausea and/or Vomiting in Patients Treated With Oxaliplatin-Based Regimens for Colorectal Cancer. J. Oncol. Pract. 8, 136–140. 10.1200/JOP.2010.000151 22942805 PMC3396799

[B18] Fujii H. Iihara H. Kajikawa N. Kobayashi R. Suzuki A. Tanaka Y. (2017). Control of Nausea Based on Risk Analysis in Patients with Esophageal and Gastric Cancer Who Received Cisplatin-based Chemotherapy. Anticancer Res. 37, 6831–6837. 10.21873/anticanres.12144 29187462

[B19] Furukawa N. Akasaka J. Shigemitsu A. Sasaki Y. Nagai A. Kawaguchi R. (2014). Evaluation of the relation between patient characteristics and the state of chemotherapy-induced nausea and vomiting in patients with gynecologic cancer receiving paclitaxel and carboplatin. Arch. Gynecol. Obstet. 289, 859–864. 10.1007/s00404-013-3058-7 24185939

[B20] Glaus A. Knipping C. Morant R. Böhme C. Lebert B. Beldermann F. (2004). Chemotherapy-induced nausea and vomiting in routine practice: a European perspective. Support. Care Cancer 12, 708–715. 10.1007/s00520-004-0662-x 15278682

[B21] Haiderali A. Menditto L. Good M. Teitelbaum A. Wegner J. (2011). Impact on daily functioning and indirect/direct costs associated with chemotherapy-induced nausea and vomiting (CINV) in a U.S. population. Support. Care Cancer 19, 843–851. 10.1007/s00520-010-0915-9 20532923

[B22] Hassan B. A. R. Yusoff Z. B. M. (2010). Negative impact of chemotherapy on breast cancer patients QOL - utility of antiemetic treatment guidelines and the role of race. Asian Pac. J. Cancer Prev. 11, 1523–1527. 21338191

[B23] Hawkins R. Grunberg S. (2009). Chemotherapy-induced nausea and vomiting: challenges and opportunities for improved patient outcomes. Clin. J. Oncol. Nurs. 13, 54–64. 10.1188/09.CJON.54-64 19193549

[B24] Hayashi T. Shimokawa M. Miyoshi T. Toriyama Y. Yokota C. Taniguchi J. (2017). A prospective, observational, multicenter study on risk factors and prophylaxis for low emetic risk chemotherapy-induced nausea and vomiting. Support. Care Cancer 25, 2707–2714. 10.1007/s00520-017-3679-7 28341971

[B25] Hayashi T. Shimokawa M. Matsuo K. Miyoshi T. Toriyama Y. Yokota C. (2018). Risk factors for delayed chemotherapy-induced nausea and vomiting with low-emetic-risk chemotherapy: a prospective, observational, multicenter study. Cancer Manage. Res. 10, 4249. 10.2147/CMAR.S176574 PMC6177523 30323680

[B26] Hesketh P. J. Aapro M. Street J. C. Carides A. D. (2010). Evaluation of risk factors predictive of nausea and vomiting with current standard-of-care antiemetic treatment: analysis of two phase III trials of aprepitant in patients receiving cisplatin-based chemotherapy. Support. Care Cancer 18, 1171–1177. 10.1007/s00520-009-0737-9 19756774

[B27] Higa G. M. Auber M. L. Hobbs G. (2012). Identification of a novel marker associated with risk for delayed chemotherapy-induced vomiting. Support. Care Cancer 20, 2803–2809. 10.1007/s00520-012-1402-2 22350597

[B28] Hilarius D. L. Kloeg P. H. van der Wall E. van den Heuvel J. J. G. Gundy C. M. Aaronson N. K. (2012). Chemotherapy-induced nausea and vomiting in daily clinical practice: a community hospital-based study. Support. Care Cancer 20, 107–117. 10.1007/s00520-010-1073-9 21258948 PMC3223596

[B29] Hu Z. Liang W. Yang Y. Keefe D. Ma Y. Zhao Y. (2016). Personalized Estimate of Chemotherapy-Induced Nausea and Vomiting: Development and External Validation of a Nomogram in Cancer Patients Receiving Highly/Moderately Emetogenic Chemotherapy. Med. (Baltimore). 95, e2476. 10.1097/MD.0000000000002476 PMC4718276 26765450

[B30] Hursti T. J. Avall-Lundqvist E. Börjeson S. Fredrikson M. Fürst C. J. Steineck G. (1996). Impact of tumour burden on chemotherapy-induced nausea and vomiting. Br. J. Cancer 74, 1114–1119. 10.1038/bjc.1996.499 8855984 PMC2077107

[B31] Iihara H. Fujii H. Yoshimi C. Yamada M. Suzuki A. Matsuhashi N. (2016). Control of chemotherapy-induced nausea in patients receiving outpatient cancer chemotherapy. Int. J. Clin. Oncol. 21, 409–418. 10.1007/s10147-015-0908-2 26475354 PMC4824820

[B32] Jones J. M. Qin R. Bardia A. Linquist B. Wolf S. Loprinzi C. L. (2011). Antiemetics for chemotherapy-induced nausea and vomiting occurring despite prophylactic antiemetic therapy. J. Palliat. Med. 14, 810–814. 10.1089/jpm.2011.0058 21554125 PMC3118930

[B33] Jordan K. Gralla R. Jahn F. Molassiotis A. (2014). International antiemetic guidelines on chemotherapy induced nausea and vomiting (CINV): content and implementation in daily routine practice. Eur. J. Pharmacol. 722, 197–202. 10.1016/j.ejphar.2013.09.073 24157984

[B34] Jordan K. Feyer P. Höller U. Link H. Wörmann B. Jahn F. (2017). Supportive Treatments for Patients with Cancer. Dtsch. Arztebl. Int. 114, 481–487. 10.3238/arztebl.2017.0481 28764837 PMC5545632

[B35] Kawazoe H. Murakami A. Yamashita M. Nishiyama K. Kobayashi-Taguchi K. Komatsu S. (2018). Patient-related Risk Factors for Nausea and Vomiting with Standard Antiemetics in Patients with Breast Cancer Receiving Anthracycline-based Chemotherapy: A Retrospective Observational Study. Clin. Ther. 40, 2170–2179. 10.1016/j.clinthera.2018.10.004 30392814

[B36] Kitazaki T. Fukuda Y. Fukahori S. Oyanagi K. Soda H. Nakamura Y. (2015). Usefulness of antiemetic therapy with aprepitant, palonosetron, and dexamethasone for lung cancer patients on cisplatin-based or carboplatin-based chemotherapy. Support. Care Cancer 23, 185–190. 10.1007/s00520-014-2339-4 25063271

[B37] Lee K.-M. Jung D.-Y. Hwang H. Kim W.-H. Lee J.-Y. Kim T.-Y. (2017). Late chronotypes are associated with neoadjuvant chemotherapy-induced nausea and vomiting in women with breast cancer. Chronobiol. Int. 34, 480–491. 10.1080/07420528.2017.1295978 28362229

[B38] Liaw C.-C. Chang H.-K. Liau C.-T. Huang J.-S. Lin Y.-C. Chen J.-S. (2003). Reduced maintenance of complete protection from emesis for women during chemotherapy cycles. Am. J. Clin. Oncol. 26, 12–15. 10.1097/00000421-200302000-00003 12576917

[B39] Mizuno M. Hiura M. Kikkawa F. Numa F. Yaegashi N. Narahara H. (2016). A prospective observational study on chemotherapy-induced nausea and vomiting (CINV) in patients with gynecologic cancer by the CINV Study Group of Japan. Gynecol. Oncol. 140, 559–564. 10.1016/j.ygyno.2015.12.029 26748216

[B40] Moher D. Liberati A. Tetzlaff J. Altman D. G. (2009). Preferred reporting items for systematic reviews and meta-analyses: the PRISMA statement. BMJ 339, b2535. 10.1136/bmj.b2535 19622551 PMC2714657

[B41] Molassiotis A. Saunders M. P. Valle J. Wilson G. Lorigan P. Wardley A. (2008). A prospective observational study of chemotherapy-related nausea and vomiting in routine practice in a UK cancer centre. Support. Care Cancer 16, 201–208. 10.1007/s00520-007-0343-7 17926070

[B42] Molassiotis A. Stamataki Z. Kontopantelis E. (2013). Development and preliminary validation of a risk prediction model for chemotherapy-related nausea and vomiting. Support. Care Cancer 21, 2759–2767. 10.1007/s00520-013-1843-2 23715816

[B43] Molassiotis A. Aapro M. Dicato M. Gascon P. Novoa S. A. Isambert N. (2014). Evaluation of risk factors predicting chemotherapy-related nausea and vomiting: Results from a European prospective observational study. J. Pain Symptom Manage. 47, 839–848. 10.1016/j.jpainsymman.2013.06.012 24075401

[B44] Molassiotis A. Lee P. H. Burke T. A. Dicato M. Gascon P. Roila F. (2016). Anticipatory Nausea, Risk Factors, and Its Impact on Chemotherapy-Induced Nausea and Vomiting: Results From the Pan European Emesis Registry Study. J. Pain Symptom Manage. 51, 987–993. 10.1016/j.jpainsymman.2015.12.317 26891606

[B45] Murakami M. Hashimoto H. Yamaguchi K. Yamaguchi I. Senba S. Siraishi T. (2014). Effectiveness of palonosetron for preventing delayed chemotherapy-induced nausea and vomiting following moderately emetogenic chemotherapy in patients with gastrointestinal cancer. Support. Care Cancer 22, 905–909. 10.1007/s00520-013-2046-6 24240649

[B46] Natale J. J. (2015). Reviewing current and emerging antiemetics for chemotherapy-induced nausea and vomiting prophylaxis. Hosp. Pract. (1995). 43 (4), 226–234. 10.1080/21548331.2015.1077095 26308912

[B47] Nawa-Nishigaki M. Kobayashi R. Suzuki A. Hirose C. Matsuoka R. Mori R. (2018). Control of Nausea and Vomiting in Patients Receiving Anthracycline/Cyclophosphamide Chemotherapy for Breast Cancer. Anticancer Res. 38, 877–884. 10.21873/anticanres.12297 29374715

[B48] NCCN (2015). Clinical Practice Guidelines in Oncology: Antiemesis, Version 2.2015. National Comprehensive Cancer Network, Available at: http://www.nccn.org/professionals/physician_gls/pdf/antiemesis.pdf [Accessed November 17, 2015].

[B49] Osoba D. Zee B. Pater J. Warr D. Latreille J. Kaizer L. (1997). Determinants of postchemotherapy nausea and vomiting in patients with cancer. Quality of Life and Symptom Control Committees of the National Cancer Institute of Canada Clinical Trials Group. J. Clin. Oncol. 15, 116–123. 10.1200/JCO.1997.15.1.116 8996132

[B50] Petrella T. Clemons M. Joy A. Young S. Callaghan W. Dranitsaris G. (2009). Identifying patients at high risk for nausea and vomiting after chemotherapy: the development of a practical validated prediction tool. II. Delayed nausea and vomiting. J. Support Oncol. 7, W9–W16.

[B51] Pollera C. F. Giannarelli D. (1989). Prognostic factors influencing cisplatin-induced emesis. Definition and validation of a predictive logistic model. Cancer 64, 1117–1122. 10.1002/1097-0142(19890901)64:5<1117::AID-CNCR2820640525>3.0.CO;2-R 2667749

[B52] Rha S. Y. Park Y. Song S. K. Lee C. E. Lee J. (2016). Controlling chemotherapy-induced nausea requires further improvement: symptom experience and risk factors among Korean patients. Support. Care Cancer 24, 3379–3389. 10.1007/s00520-016-3146-x 26984242

[B53] Roscoe J. A. Bushunow P. Morrow G. R. Hickok J. T. Kuebler P. J. Jacobs A. (2004). Patient expectation is a strong predictor of severe nausea after chemotherapy: a University of Rochester Community Clinical Oncology Program study of patients with breast carcinoma. Cancer 101, 2701–2708. 10.1002/cncr.20718 15517574

[B54] Roscoe J. A. Morrow G. R. Colagiuri B. Heckler C. E. Pudlo B. D. Colman L. (2010). Insight in the prediction of chemotherapy-induced nausea. Support. Care Cancer 18, 869–876. 10.1007/s00520-009-0723-2 19701781 PMC3017350

[B55] Sekine I. Segawa Y. Kubota K. Saeki T. (2013). Risk factors of chemotherapy-induced nausea and vomiting: Index for personalized antiemetic prophylaxis. Cancer Sci. 104, 711–717. 10.1111/cas.12146 23480814 PMC7657206

[B56] Shih V. Wan H. S. Chan A. (2009). Clinical predictors of chemotherapy-induced nausea and vomiting in breast cancer patients receiving adjuvant doxorubicin and cyclophosphamide. Ann. Pharmacother. 43, 444–452. 10.1345/aph.1L437 19193584

[B57] Shimokawa M. Hayashi T. Kogawa T. Matsui R. Mizuno M. Kikkawa F. (2019). Evaluation of Combination Antiemetic Therapy on CINV in Patients With Gynecologic Cancer Receiving TC Chemotherapy. Anticancer Res. 39, 225–230. 10.21873/anticanres.13101 30591462

[B58] Sugino S. Janicki P. K. (2015). Pharmacogenetics of chemotherapy-induced nausea and vomiting. Pharmacogenomics 16, 149–160. 10.2217/pgs.14.168 25616101

[B59] Sun C. C. Bodurka D. C. Weaver C. B. Rasu R. Wolf J. K. Bevers M. W. (2005). Rankings and symptom assessments of side effects from chemotherapy: insights from experienced patients with ovarian cancer. Support. Care Cancer 13, 219–227. 10.1007/s00520-004-0710-6 15538640

[B60] Takemoto H. Nishimura J. Komori T. Kim H. M. Ota H. Suzuki R. (2017). Combination antiemetic therapy with aprepitant/fosaprepitant in patients with colorectal cancer receiving oxaliplatin-based chemotherapy in the SENRI trial: analysis of risk factors for vomiting and nausea. Int. J. Clin. Oncol. 22, 88–95. 10.1007/s10147-016-1022-9 27465476

[B61] Tamura K. Aiba K. Saeki T. Nakanishi Y. Kamura T. Baba H. (2015). Testing the effectiveness of antiemetic guidelines: results of a prospective registry by the CINV Study Group of Japan. Int. J. Clin. Oncol. 20 (5), 855–865. 10.1007/s10147-015-0786-7 25681876

[B62] Tsuji Y. Baba H. Takeda K. Kobayashi M. Oki E. Gotoh M. (2017). Chemotherapy-induced nausea and vomiting (CINV) in 190 colorectal cancer patients: a prospective registration study by the CINV study group of Japan. Expert Opin. Pharmacother. 18, 753–758. 10.1080/14656566.2017.1317746 28395603

[B63] Tsuji D. Suzuki K. Kawasaki Y. Goto K. Matsui R. Seki N. (2019). Risk factors associated with chemotherapy-induced nausea and vomiting in the triplet antiemetic regimen including palonosetron or granisetron for cisplatin-based chemotherapy: analysis of a randomized, double-blind controlled trial. Support. Care Cancer 27, 1139–1147. 10.1007/s00520-018-4403-y 30094732

[B64] Uchida M. Mori Y. Nakamura T. Kato K. Kamezaki K. Takenaka K. (2017). Comparison between Antiemetic Effects of Palonosetron and Granisetron on Chemotherapy-Induced Nausea and Vomiting in Japanese Patients Treated with R-CHOP. Biol. Pharm. Bull. 40, 1499–1505. 10.1248/bpb.b17-00318 28867732

[B65] Warr D. G. Street J. C. Carides A. D. (2011). Evaluation of risk factors predictive of nausea and vomiting with current standard-of-care antiemetic treatment: analysis of phase 3 trial of aprepitant in patients receiving adriamycin-cyclophosphamide-based chemotherapy. Support. Care Cancer 19, 807–813. 10.1007/s00520-010-0899-5 20461438

[B66] Warr D. (2014). Prognostic factors for chemotherapy induced nausea and vomiting. Eur. J. Pharmacol. 722, 192–196. 10.1016/j.ejphar.2013.10.015 24157977

[B67] Yap K. Y.-L. Low X. H. Chui W. K. Chan A. (2012). Computational prediction of state anxiety in Asian patients with cancer susceptible to chemotherapy-induced nausea and vomiting. J. Clin. Psychopharmacol. 32, 207–217. 10.1097/JCP.0b013e31824888a1 22367655

